# The role of serum levels of vitamin D in children's muscle strength: A systematic review

**DOI:** 10.6061/clinics/2021/e3200

**Published:** 2021-09-06

**Authors:** Ana B.J. da Silva, Taciane S. do Carmo, Ana P.S. Souza, Mariluce R.M. Silva, Matheus S.S. Fernandes, Viviane O.N. Souza, Waleska M.A. Barros

**Affiliations:** I Programa de Pos-graduacao em Neuropsiquiatria e Ciencias do Comportamento, Universidade Federal de Pernambuco, Recife, PE, BR.; II Centro Universitario Facol / Centro Integrado de Tecnologias em Neurociencia (CITENC), Vitoria de Santo Antao, PE, BR.; III Universidade Federal de Pernambuco, Centro Academico Vitoria, Vitoria de Santo Antao, PE, BR.

**Keywords:** Vitamin D, Muscle Strength, Child, Hand Strength

## Abstract

This review aims to investigate the different levels of vitamin D and its role in muscle strength in healthy children and non-athletes. A search conducted in three databases (PubMed, Scopus, and Psycinfo) resulted in 655 articles, which were systematically analyzed and selected based on the following criteria: (a) original cross-sectional studies and clinical trials; (b) healthy children aged 5-11 years; (c) no language restriction or year of publication; and (d) studies that assessed the possible relationship between vitamin D levels and muscle strength. Six studies were included because they met all the inclusion criteria. According to the findings of this review, factors such as sex, skin color, and vitamin D supplementation early in life modulate the levels of vitamin D in the body, and there is a relationship between muscle strength and vitamin D levels. Interestingly, vitamin D supplementation is not always significantly associated with increased muscle grip strength. However, there is a scarcity of studies that aim to analyze the possible effects of different levels of vitamin D on muscle function and neuromuscular variables in physically inactive children and non-athletes without previously diagnosed disease. Further studies are warranted in the future to address the gap in the literature.

## INTRODUCTION

Serum levels of vitamin D may suffer variability due to genetic and environmental factors related to general metabolism, resulting in changes in its integumentary synthesis and bioavailability (1,2). In addition, maintaining physiological levels of vitamin D is essential for bone quality and vitamin D deficit can reduce intestinal calcium absorption, decrease bone mineralization levels, and induce osteomalacia (3-5). The effects of vitamin D in the human body have been increasingly studied, including its role in regulating gene expression in the small intestine, immune system, and epidermal, cardiovascular, and neuromuscular tissues, among others (1,6,7). In this sense, vitamin D receptors (VDRs) have been found in peripheral tissues as well as in skeletal muscle (8), prompting the exploration of possible associations of vitamin D levels with muscle function.

Vitamin D can increase the capacity to produce strength through the readiness of type II muscle fibers (9) and favor the stimulation of protein synthesis in regions containing these fibers (10). These processes are activated by metabolites produced in the muscle tissue, including calcitriol (1,25(OH)_2_D) (11), the biologically active form of vitamin D. Calcitriol modulates muscle contractility through the generation of second messengers (12) and intra-and extracellular regulation of calcium ions, the latter of which is capable of stimulating the secretion of parathyroid hormone (13). Thus, vitamin D is critical in muscle function, suggesting it may influence muscle strength and muscle contraction rate.

Palacios and Gonzales (14) reported that globally, low levels of vitamin D are a problem affecting all age groups, particularly, vitamin D insufficiency affects babies, children, and adolescents in countries in South America and Africa. In addition, the effects of different levels of vitamin D and supplementation on bone health (15), athletic performance, various pathological conditions in children (16,17), including acute respiratory tract infections (18), the risk of sleep disorders (19), and the association with oxidative stress and inflammation (20) were investigated. There have been relatively few studies of the possible effects of different levels of vitamin D on muscle function and neuromuscular variables in physically inactive children and non-athletes without previously diagnosed disease. The objective of this review was to investigate the effects of different levels of vitamin D and role of vitamin D in muscle strength in healthy children and non-athletes.

## MATERIALS AND METHODS

**Figure 1:**
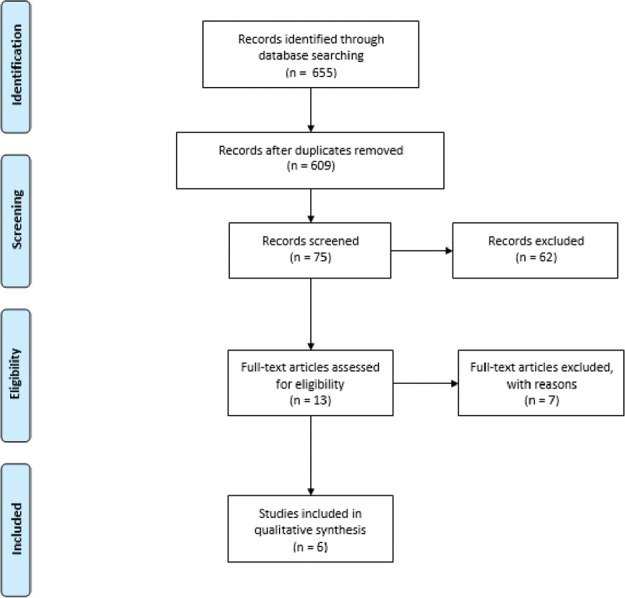
Flowchart of bibliographic research and selection of studies for this systematic review according to PRISMA.

This systematic review adopted the criteria recommended by the Preferred Reporting Items for Systematic Reviews and Meta-Analyses (PRISMA) method (Figure 1), which was registered on 01/01/2021 in the International Prospective Register of Systematic database Reviews (PROSPERO) with registration number CRD42021223896.

### Search strategy

The following databases were used to perform our searches: PubMed (MEDLINE), Scopus, and Psycinfo with the following combination of terms contained in the Medical Subject Headings: “Vitamin D” AND “Muscle Strength” OR “Strength, Muscle” and “Child”.

### Selection of studies and inclusion / exclusion criteria

Two independent researchers (ABJ and TS) read the study titles and abstracts and, if necessary, read the entire text. In case of disagreement, a third researcher was consulted to establish a consensus. The following inclusion criteria were established: (a) original cross-sectional studies and clinical trials; (b) healthy children aged 5-11 years; (c) no language restriction or year of publication; and (d) studies that assessed the possible relationship between vitamin D levels and muscle strength. Studies that included child athletes, carriers of some diseases, or children receiving maintenance drug therapy were excluded. In addition, the Population Intervention Comparator Outcome strategy was used to select studies with greater specificity.

### Data extraction

ABJ and TS independently reviewed the selected articles to extract relevant data for the preparation of this review. Conflicts of opinion between the authors were mitigated by a third reviewer. It is important to note that searches performed using electronic databases were performed without the aid of any software.

### Risk of bias

**Figure 2:**
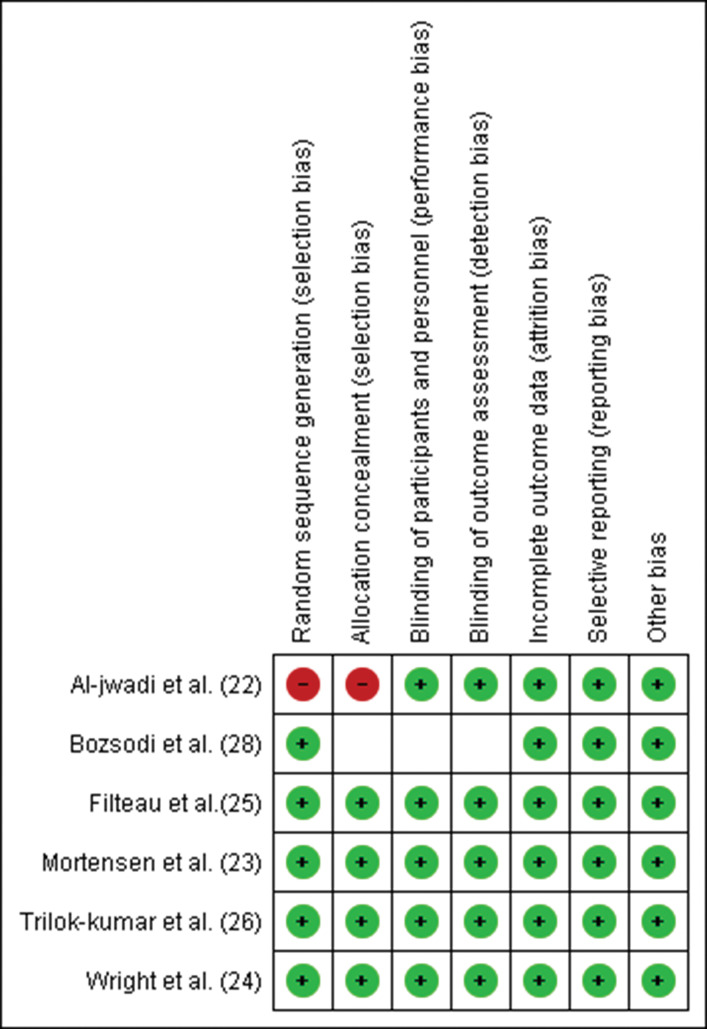
Risk of bias summary: review authors’ judgements about each risk of bias item for each included study.

**Figure 3:**
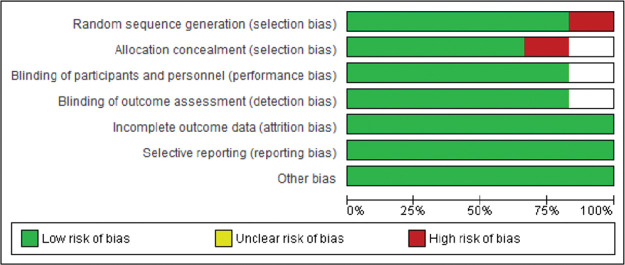
Risk of bias graph: review authors’ judgements about each risk of bias item presented as percentages across all included studies.

The Joanna Briggs Institute (JBI) tool (21) was used to assess the quality of the included studies. Each study was categorized according to the percentage of positive responses to the JBI questions. As a complementary analysis of the risk of bias, Review Manager (RevMan), Version 5.3.0 was used to detect intervening factors from the seven judgment criteria provided by the software: (I) random sequence generation, (II) allocation concealment, (III) blinding of participants and personnel, (IV) blinding of outcome assessment, (V) incomplete outcome data, (VI) selective reporting, and (VII) other bias. Thus, the present review presents a low risk of bias, as shown in Figures 2 and 3.

## RESULTS

A total of 655 titles and 88 abstracts were analyzed. Eighty-two articles were excluded because they did not meet the eligibility criteria or because they were duplicates. A total of 262 studies were excluded because they included unhealthy individuals, which constituted the main cause of exclusion. Four cross-sectional studies and two clinical trials were included. The flowchart for the selection and inclusion of articles is shown in Figure 1 (PRISMA).

### Characteristics of the studies

The study participants included in this review were located in different geographic locations, including North America, Europe, and Asia. The age range was 5-11 years. Three studies were identified with insufficient classification of vitamin D levels (22-24), and two studies (25,26) included subjects with deficient vitamin D levels as defined by the International Life Science Institute of Brazil (27). The characteristics of the included studies are listed in Table 1.

## MAIN OUTCOME

It is important to note that vitamin D3 undergoes enzymatic conversion in the liver by vitamin D-25-hydroxylase to produce 25-hydroxyvitamin D (25OHD). 25OHD is the main circulating form of vitamin D in the body.

Possible relationships between handgrip strength (HGS) and vitamin D levels were analyzed in all studies, as shown in Table 2. In one study (28), serum vitamin D levels were not evaluated, although variants of the VDR gene were analyzed in relation to HGS. Wright et al. (22) presented data that took into account the subjects' race and sex. In this study, white girls had higher levels of circulating 25OHD (80.5±6.1 nmol/L) than black girls (58.9±18.2 nmol/L) and white and black boys (79.8±3.0 nmol/L and 62.0±15.2 nmol/L, respectively). In relation to HGS, white girls showed less capacity to produce strength (148.08 N±77.47 N) compared with black girls (202.02±85.31 N) and black and white boys (223.59±70.60 N and 159.85±83.35 N). However, following vitamin D3 supplementation for 12 weeks, there were significant changes in HGS (1.8%±123.2%) and 25OHD levels (34.9%±55.9%); however, the HGS was not related to changes in 25OHD levels after vitamin D supplementation.

Filteau et al. (25) analyzed the possible association of HGS with supplementation of 25OHD during the first 6 months of life in children aged 5 years. They found that 43% of the children assessed had insufficient serum levels of 25OHD, and 40% had borderline serum levels of 25OHD. The average serum 25OHD level was 32.7±23.0 nmol/L. Children in this study had an average HGS of 24.41±9.12 N. The authors concluded that there was a weak association between the levels of 25OHD and HGS (25OHD [log ng/mL]=0.005[-0.23 to 0.24], *p*=0.96)

Trilok-Kumar et al. (26) studied the long-term effects of vitamin D supplementation using participants who were supplemented with vitamin D at 6 months of age in a previous study (29). They demonstrated that there were no significant differences between the supplementation and placebo groups in the children's performance in motor tests or HGS assessment. The HGS was 24.71±8.72 N for the group that received vitamin D supplementation and 24.02±9.51 N for the placebo group.

To determine whether muscle strength is associated with the plasma levels of 25OHD, Mortensen et al. (23) analyzed 130 Danish white children who received vitamin D3 supplementation for 20 weeks. During this intervention, doses of 0, 10, or 20 μg/day were supplemented, giving rise to three different groups. Associations between muscle strength and the plasma levels of 25OHD were found through analyses stratified by sex before supplementation. A positive association between 25OHD and muscle strength (HGS) was observed in female children (*p*=0.005). After supplementation, all children had vitamin D levels ≥50 nmol/L and muscle strength did not differ between groups. Before supplementation, the HGS was 92.18±4.9 N (placebo group), 107.87±5.88 N (10 μg/day) and 106.89±4.90 (20 μg/day). After supplementation, the HGS was 98.06±5.88 N (placebo group), 112.78±5.88 N (10 μg/day) and 109.83±4.90 N (20 μg/day). In this study, there was no difference between the sexes in terms of muscle strength.

Al-jwadi et al. (24) found that serum 25OHD values were positively associated with HGS in both sexes (β=0.008, 95% confidence interval [CI]: 0.002, 0.014, *p*=0.013). However, in girls, there was a significant association (β=0.009, 95% CI: 0.001, 0.018; *p*=0.031), demonstrating an increase in serum 25OHD of 25 nmol/L resulted in an increase in HGS of 2.25 N. It is worth mentioning that the mean HGS value for these children was 82.76±16.96 N.

Bozsodi et al. (28) determined the effect of VDR genotype on HGS and identified six VDR single nucleotide polymorphisms (SNPs): rs4516035 (A1012G), rs2228570 (Fokl), rs3782905 (Ddel), rs1544410 (Bsml), rs731236 (Taql), and rs10783215. A significant association was observed between three SNPs (A012G, Bsml, and Taql) and HGS; the combination of “TT” alleles of the A1012G gene was related to higher levels of HGS, while the “CC” genotype was related to lower HGS levels. An increase in HGS of the dominant hand was related to the Bsml genotype “AA” (*p*=0.010) and Taql genotype “CC” (*p*=0.038). In addition, three VDR haplotypes were significantly associated with HGS in the dominant and non-dominant hands (*p*<0.005 and *p*<0.01, respectively).

## DISCUSSION

This review investigated scientific literature concerning the relationship between serum vitamin D levels and muscle strength in healthy children. In general, the studies included in this review observed a relationship between muscle strength, measured through HGS, and the serum levels of 25OHD. Variables such as sex, age, and ethnicity can be intervening factors in this relationship. Furthermore, it was found that vitamin D supplementation was not always significantly associated with an increase in HGS, suggesting the need for further studies to consolidate results on this association.

It is important to note that none of the studies included in this review (selected according to our inclusion criteria) were executed in countries with a low Human Development Index nor in countries with a tropical climate. Both are important sociogeographic factors that directly influence an individual's serum vitamin D levels, environmentally and with regard to vitamin D synthesis (30).

HGS was used as a predictor of motor performance in healthy children in all studies included in this review. HGS is favorable among muscle function tests because it is simple, non-invasive (31), and inexpensive. During growth and development, changes in skeletal muscle structure and strength are accompanied by simultaneous changes in bone tissue (32), establishing a close relationship between these tissues. Studies excluded from this review have identified an association between vitamin D levels and HGS in children (33-35) and observed a positive relationship between these variables. Wakayo et al. (35) conducted a study of 174 Ethiopian schoolchildren from urban and rural regions in 2013 to verify the association of vitamin D and HGS. They reported a mean serum 25OHD level of 54.5±15.8 nmol/L and mean HGF of 172.6±67.66 N, indicating a positive correlation between the serum level of vitamin D and HGS, although this correlation was not statistically significant (r=0.087; *p*=0.256).

A systematic review conducted in 2019 (1) reported factors that may influence the vitamin D levels in the human body. One influential factor is the synthesis that occurs in the skin that is influenced by the use of sunscreen and skin pigmentation. The latter reduces the production of vitamin D by up to 99%. The synthesis of vitamin D and, consequently, serum vitamin D levels are strongly influenced by the amount of melanin present in the assessed individual. Melanin has two chemical forms: eumelanin and pheomelanin. Eumelanin is more efficient than pheomelanin in blocking ultraviolet (UV) photons, and lighter-skinned people are more sensitive to these rays (36). Humans can obtain vitamin D in two ways: diet and sun exposure. In the latter, UV-B radiation penetrates the skin and converts pre-vitamin D3 into vitamin D (2).

One of the studies included in this review (22) presented data that considered the subjects' skin color and gender. In this study, white girls had higher levels of circulating 25OHD (80.5±6.1 nmol/L) than black girls (58.9±18.2 nmol/L) and white and black boys (79.8±3.0 nmol/L and 62.0±15.2 nmol/L, respectively). However, in relation to HGS, white girls had less capacity to produce strength (148.08±77.47 N) compared with black girls (202.02±85.31 N) and black and white boys (223.59±70.60 N and 159.85±83.35 N, respectively). When vitamin D3 supplementation was administered for 12 weeks, there was a change in the production capacity of HGS (1.8%±123.2%) and 25OHD levels (34.9%±55.9%). However, HGS was not related to changes in 25OHD levels following intervention with supplementation.

Regarding the influence of sex, changes in HGS related to 25OHD levels were observed in children of both sexes. However, only female individuals showed a positive and significant association (24,31). In contrast, Carson et al. (37) performed linear regression analysis and observed that the standardized serum concentration of 25OHD was positively associated with muscle strength in boys aged 15 years regardless of pubertal status, physical activity, year, and energy-adjusted protein intake (*p*<0.001). However, it is necessary to better explain the differences between the sexes to determine the possible relationship between HGS and vitamin D levels from this perspective.

According to our results, supplementation with vitamin D can increase serum 25OHD levels, although this change does not appear to be related to increases in HGS. Studies conducted in children with health impairments have reported the effects of vitamin D supplementation on muscle strength (38-40) and concluded that vitamin D supplementation, in combination with a training program, significantly increased muscle strength in children with severe burns. High doses of vitamin D can improve neuromuscular motor skills in HIV-positive children. In addition to improving physical performance, vitamin D supplementation also improved the quality of life of children with sickle cell disease.

Although there are discrepancies regarding the association between vitamin D and muscle strength, studies conducted in a population with some motor limitations or in individuals with deficient or insufficient 25OHD levels showed significant positive effects of vitamin D supplementation on neuromuscular abilities (34,38,41). In addition, studies in animal models (42-44) have shown an association between muscle strength and vitamin D levels in conditions of vitamin D deficiency and insufficient levels of vitamin D and/or associated or pre-existing disease.

Vitamin D deficits in childhood can result in physical and mental health problems in adulthood and old age. One of the many variables that influence serum levels of vitamin D is ethnicity. Some reviews point out that cultural effects may cause vitamin D deficiency, often severe, due to factors such as clothing, beliefs, eating habits, and even the levels of physical activity (45-47). The data gathered in this review illustrate the importance and possible modulation of vitamin D levels and its relationship to muscle strength in children However, this review also reveals the need for further studies on vitamin D levels in non-athlete children without comorbidities and the consequences of vitamin D levels muscle function throughout their lives.

## AUTHOR CONTRIBUTIONS

Silva ABJ and Carmo TS contributed to the research conception, data collection, interpretation of results, and critical review of the manuscript. Souza APS, Silva MRM, and Fernandes MSS contributed to data analysis and interpretation, manuscript drafting and critical review. Souza VON and Barros WMA contributed to the data collection and critical review of the manuscript.

## Figures and Tables

**Table 1:** Descriptions of the studies included in this systematic review: author and year, country, type of study, number of participants, sample size and age of participants, and sex.

Author, year	Country	Type of study	Number of participants	Sample size, n/age, years	Sex	
					Female	Male
Wright et al. (22)	United States of America	Clinical trial	324	323/11.3±1.2	162	162
Bozsodi et al. (28)	Hungary	Cross-sectional study	706	706/9.8±1.2	346	360
Filteau et al. (25)	India	Cross-sectional study	912	902/5.0±1.0	478	434
Mortensen et al. (23)	Denmark	Clinical trial	130	130/6.6±1.5	69	61
Trilok-Kumar et al. (26)	India	Cross-sectional study	912	912/5.0±1.0	475	437
Al-Jwadi et al. (24)	Denmark	Observational study	881	881/5.0±0.06	432	449

**Table 2:** Descriptions of the studies included in the systematic review: author and year, measurement of vitamin D and muscle strength, vitamin D level results, vitamin D supplementation, and muscle strength status.

Author, year	Measurement		Vitamin D level results	Vitamin D supplementation	Muscle strength status
	Vitamin D	Muscle strength			
Wright et al. (22)	Blood samples after an overnight fast. Serum 25OHD was evaluated using a two-step radioimmunoassay.	Digital dynamometer.	Before intervention in 318 participants: 69.9±18.5 nmol/L. After intervention of vitamin D of 12 weeks: Δ 22.37±32.99 nmol/L.	Oral dose of vitamin D3 of 0, 400, 1000 or 4000 IU/day. Time: 12 weeks.	Handgrip strength before intervention in 288 participants: 176.52±93.16 N. After 12-week intervention: Δ 7.25±25.49 N.
Bozsodi et al. (28)	For genotyping, saliva samples were analyzed using the Orange OG-500 kit.	-	Six candidates for single nucleotide polymorphisms were identified: A1012G, Fokl, Ddel, Bsml, Taql, and rs10783215.	Without intervention.	Handgrip strength in 706 participants: dominant hand strength 147.5±42.1 N.
Filteau et al. (25)	Radioimmunoassay duplicate using a DiaSorin kit and an external standard (vitamin D external Quality Assessment Scheme, DEQAS).	Custom dynamometer.	902 participants: 32.7±23.0 nmol/L.	Without intervention.	Handgrip strength in 830 participants: 24.41±9.12 N.
Mortensen et al. (23)	A 25 mL venous sample of blood was collected after 2 to 4h of fasting. The 25OHD serum was analyzed by tandem mass spectrometry liquid chromatography.	Manual dynamometer.	117 participants: 56.1±12.8 nmol/L. Placebo group before the intervention: 55.6±1.7 nmol/L. After intervention: 31.4±1.2 nmol/L The 10 μg/day group before the intervention: 56.9±2.1 nmol/L. After intervention: 61.8±1.7 nmol/L. The 20 μg/day group before the intervention: 58.6±2.1 nmol/L After intervention: 76.0±1.8 nmol/L.	Vitamin D_3_ supplementation was performed for 20 weeks. Subjects were divided into three groups: Placebo: 40 subjects. 10 μg/day: 38 individuals. 20 μg/day: 39 individuals.	Mean peak strength for the placebo group before the intervention: 92.18±4.9 N. After intervention: 98.06±5.88 N. 10 μg/day before the intervention: 107.87±5.88 N. After intervention: 112.78±5.88 N. 20 μg/day before the intervention: 106.89±4.90 N. After intervention: 109.83±4.90 N.
Trilok-Kumar et al. (26)	Serum OHD duplicate in radioimmunoassay using a Diasorin kit. An external standard (DEQAS).	Custom design dynamometer.	436 participants who supplemented vitamin D early in life: 32.0 nmol/L. 461 participants in the placebo group: 33.6 nmol/L.	Without intervention.	Early intervention group of vitamin D in 405 participants: 24.71±8.72 N. Placebo group 425 participants: 24.02±9.51 N.
Al-Jwadi et al. (24)	25OHD serum levels were analyzed by liquid chromatography mass spectrometry.	Digital hand Dynamometer.	499 participants: 70.73±24.48 nmol/L.	Without intervention.	881 participants: 82.76±16.96 N.

Caption: 25(OH)D: 25-hydroxyvitamin D.
